# Standard mouse diets lead to differences in severity in infectious and non-infectious colitis

**DOI:** 10.1128/mbio.03302-24

**Published:** 2025-03-24

**Authors:** Joshua E. Denny, Julia N. Flores, Nontokozo V. Mdluli, Michael C. Abt

**Affiliations:** 1 Department of Microbiology, Perelman School of Medicine, University of Pennsylvania, Philadelphia, Pennsylvania, USA; 2 Division of Infectious Disease, Department of Pediatrics, The Children’s Hospital of Philadelphia, Philadelphia, Pennsylvania, USA; 3 Institute for Immunology and Immune Health, Perelman School of Medicine, University of Pennsylvania, Philadelphia, Pennsylvania, USA; University of Wisconsin-Madison, Madison, Wisconsin, USA

**Keywords:** *Clostridioides difficile*, diet, gut inflammation, colitis, gut microbiota, metabolome

## Abstract

*Clostridioides difficile* infects the large intestine and can result in debilitating and potentially fatal colitis. The intestinal microbiota is a major factor influencing the severity of disease following infection. Factors like diet that shape microbiota composition and function may modulate *C. difficile* colitis. Here, we report that mice fed two distinct standard mouse chows (LabDiet 5010 and LabDiet 5053) exhibited significantly different susceptibility to severe *C. difficile* infection. Both diets are grain-based with comparable profiles of macro and micronutrient composition. Diet 5010-fed mice had severe morbidity and mortality compared to Diet 5053-fed mice despite no differences in *C. difficile* colonization or toxin production. Furthermore, Diet 5053 protected mice from toxin-induced epithelial damage. This protection was microbiota-dependent as germ-free mice or mice harboring a reduced diversity microbiota fed Diet 5053 were not protected from severe infection. However, cohousing with mice harboring a complex microbiota restored the protective capacity of Diet 5053 but not Diet 5010. Metabolomic profiling revealed distinct metabolic capacities between Diet 5010- and Diet 5053-fed intestinal microbiotas. Diet 5053-mediated protection extended beyond *C. difficile* infection as Diet 5053-fed mice displayed less severe dextran sodium sulfate-induced colitis than Diet 5010-fed mice, highlighting a potentially broader capacity for Diet 5053 to limit colitis. These findings demonstrate that standard diet formulations in combination with the host microbiota can drive variability in severity of infectious and non-infectious murine colitis systems, and that diet holds therapeutic potential to limit the severity of *C. difficile* infection through modulating the functional capacity of the microbiota.

**IMPORTANCE:**

Diet is a major modulator of the microbiota and intestinal health. This report finds that two different standard mouse diets starkly alter the severity of colitis observed in a pathogen-mediated (*Clostridioides difficile*) and non-infectious (dextran sodium sulfate) mouse colitis experimental systems. These findings in part explain study-to-study variability using these mouse systems to study disease. Since the gut microbiota plays a key role in intestinal homeostasis, diet-derived modulation of the microbiota is a promising avenue to control disease driven by intestinal inflammation and may represent a potential intervention strategy for at-risk patients.

## INTRODUCTION

*Clostridioides difficile* is an urgent public health threat due to its prevalence in the nosocomial setting and high rate of recurrence (1). Infection severity can range from mild diarrhea to pseudomembranous colitis, toxic megacolon, and death (2, 3). The rise of antibiotic resistance in *C. difficile* (4, 5), along with its status as one of the most common hospital-acquired infections globally, highlights the need for new treatments (1, 6, 7). One of the largest risk factors for infection is broad-spectrum antibiotic use, which disrupts the intestinal microbiota and decreases colonization resistance to *C. difficile* (8, 9). Recent research has focused on microbiota-based therapies as a novel way to treat infection. Diet, as one of the major determinants of microbiota composition and function (10), is a promising avenue to pursue for modulating the microbiota for therapeutic purposes. Previous work has found that dietary components can modulate the severity of *C. difficile* infection. For example, the aryl hydrocarbon receptor agonist indole-3-carbinol, a microbiota-derived byproduct of cruciferous vegetables, and low-protein diets are protective following infection (11, 12), while high-fat and high-protein diets can enhance *C. difficile* virulence (13–15). Similarly, dietary trehalose or sialic acid from the breakdown of mucus can act as carbon sources to enhance *C. difficile* virulence (16, 17), while the fiber inulin and resulting short-chain fatty acids are associated with suppressing *C. difficile* fitness (18). Taken together, there is potential for diet-mediated therapies for modulating *C. difficile* infection.

The impact of diet on other types of intestinal inflammation such as inflammatory bowel disease (IBD) has also been investigated. Specific diets like the Mediterranean diet, the specific carbohydrate diet, or the low FODMAP diet can mitigate the severity and frequency of IBD flares by reducing the availability of complex carbohydrates that can be fermented by commensals or used by pro-inflammatory bacteria as an energy source (19, 20). In more severe cases, Crohn’s disease patients who fail biologics can resort to a diet that is a combination of partial enteral nutrition supplementation and a strict exclusion diet for relief from intestinal symptoms (21–23). Conversely, high-fat diets and high-protein diets are positively correlated with more severe colitis (20). Therefore, dietary composition is an essential factor to consider for any study investigating the etiology of colitis.

The severity of *C. difficile* infection is dependent on multiple factors, with some of the largest contributors being the strain of *C. difficile*, the host immune response, and the intestinal microbiota. The use of mice as a model system to study *C. difficile* pathogenesis was developed in 2008 (24). Since then, mouse studies have provided significant advances in our understanding of how host immunity, commensal bacteria, and *C. difficile* interact during disease. However, studies using this infection system have reported variability in the severity of disease experienced by mice that cannot be completely explained despite using the same mouse strains, *C. difficile* strains, or antibiotic regimens prior to infection (22). These fluctuations in disease severity can hamper the ability to compare results between reports and hinder progress in the field. Our group has observed differences in *C. difficile* infection severity in wild-type mice from two different mouse vivaria despite using identical infection protocols, mouse strains, and *C. difficile* strains (25, 26). In this report, we determined a source of disparate disease phenotypes to be the different standard mouse chow diets used in each vivarium. Mice fed Purina LabDiet 5010 (Diet 5010) exhibited severe disease following *C. difficile* infection, while mice receiving Purina LabDiet 5053 (Diet 5053, also known as PicoLab Rodent Diet 20) had significantly reduced disease severity. Both diets are grain-based with similar nutrient profiles in terms of protein, fiber, fat, and vitamin composition. The protection afforded by Diet 5053 was microbiota-dependent and was not unique to *C. difficile* infection, as Diet 5053-fed mice treated with dextran sodium sulfate (DSS) also exhibited less severe colitis compared to Diet 5010-fed mice.

## RESULTS

### Mice fed two different standard mouse diets have different *C. difficile* infection outcomes

**Fig 1:**
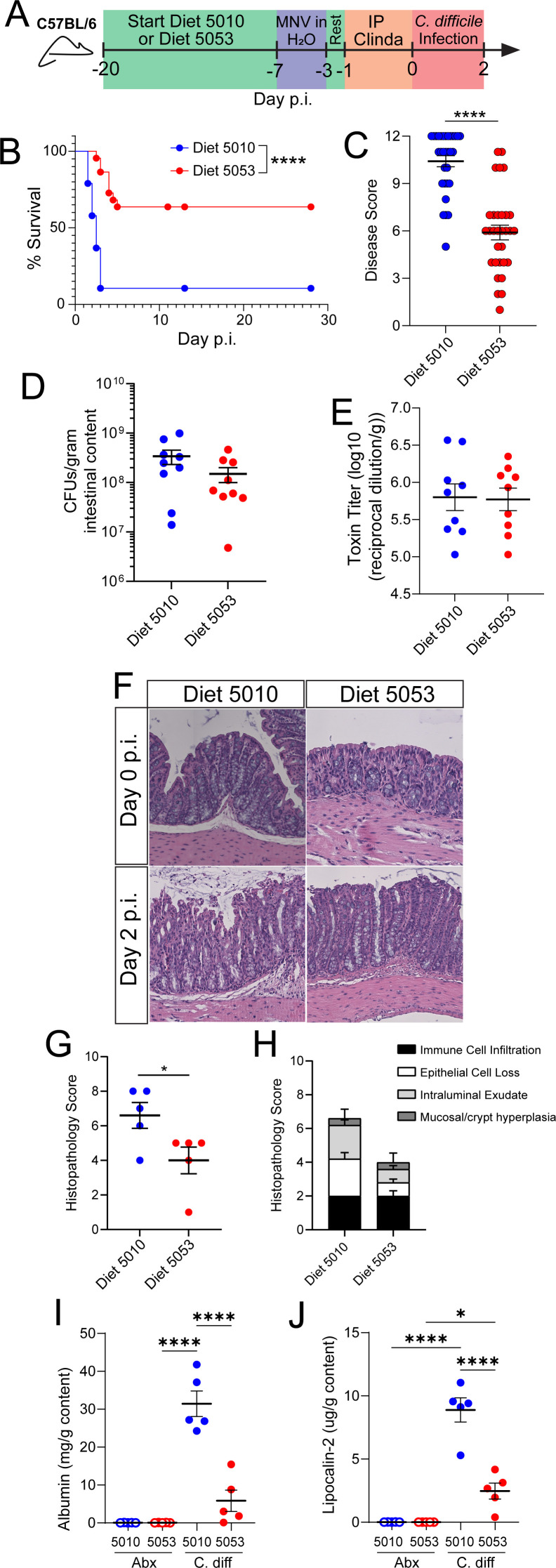
Different standard mouse diets lead to differential *C. difficile* infection outcomes. (A) Experimental design of *C. difficile* infection. (B) Survival of mice fed Diet 5010 or Diet 5053. Diet 5010 (*n* = 19), Diet 5053 (*n* = 22). (C) Disease severity score at day 2 p.i. between mice fed Diet 5010 or Diet 5053. Diet 5010 (*n* = 31), Diet 5053 (*n* = 31). (D) CFU of *C. difficile* in the intestinal content at day 2 p.i. Diet 5010 (*n* = 16), Diet 5053 (*n* = 17). (E) Total toxin concentration in the large intestine at day 2 p.i. Diet 5010 (*n* = 9), Diet 5053 (*n* = 9). (F) Representative hematoxylin and eosin staining of proximal colon tissue before and during *C. difficile* infection. (G) Histopathological composite scoring of mice fed Diet 5010 and Diet 5053 and infected with *C. difficile*. Diet 5010 (*n* = 5), Diet 5053 (*n* = 5). (H) Breakdown of scoring seen in panel G. Scoring: scale of unremarkable (0) to severe (4) in the categories immune cell infiltration, epithelial cell loss, crypt hyperplasia, and intraluminal exudate. Diet 5010 (*n* = 5), Diet 5053 (*n* = 5). (I) Albumin concentration from luminal intestinal content at day 2 p.i.; for all groups, *n* = 5. (J) Lipocalin-2 concentration from luminal intestinal content at day 2 p.i.; for all groups, *n* = 5. Data are mean ± SEM. Data in panel B were analyzed by the Mantel-Cox (log rank) test; data in panels C–E, G, I, and J were analyzed by *t*-test. * = *P* < 0.05; **** = *P* < 0.0001.

The murine *C. difficile* infection model used here mimics the main route of exposure in humans following antibiotic treatment, which is the primary risk factor for infection (24). Although this model is widely used, the severity of disease experienced by mice can vary greatly despite the same genetic background, antibiotic treatment, or strain of *C. difficile* used (27–29). Previous reports from our groups have observed that C57BL/6 mice infected with the same strain of *C. difficile* and the same infection protocol in two different vivaria experience differences in disease severity (25, 26). While multiple factors can vary between institutions and vivaria such as water source, bedding, facility-specific commensal bacteria, and husbandry protocols (30–34), one notable difference between the two vivaria in question was the mouse chow used, LabDiet 5010 or LabDiet 5053. Both Diet 5010 and Diet 5053 are grain-based and categorized as “standard mouse chow” with comparable macronutrient compositions (Table S1) (35, 36). To determine if these different diets contributed to the disparate disease phenotypes and control for inter-vivarium factors, C57BL/6 mice bred and weaned in one vivarium were fed the two different diets starting at 8 weeks of age and put through the same *C. difficile* infection protocol. Mice were treated with metronidazole, neomycin, vancomycin (MNV) in the drinking water for 4 days, switched to regular drinking water, given a single dose injection of clindamycin intraperitoneally 1 day prior to infection, and then inoculated with spores (VPI10463 strain) by oral gavage (Fig. 1A). The two different mouse chows led to significant differences in *C. difficile* infection severity, with Diet 5010-fed mice exhibiting severe disease, while Diet 5053-fed mice had significantly less mortality (Fig. 1B) and lower peak disease score at day 2 post-infection (p.i.) (Fig. 1C). While both groups of mice had similar weight loss (Fig. S1A), the increased disease score in Diet 5010-fed mice was driven by loss of body temperature at day 2 p.i. (Fig. S1B). Similarly, Diet 5053-fed mice had significantly lower diarrhea and morbidity scores (Fig. S1C and D) compared to Diet 5010-fed mice. To determine if this diet-mediated effect was strain-specific, C57BL/6 mice were also infected with *C. difficile* strain R20291 (Ribotype 027). Diet 5053-fed mice were also more protected compared to Diet 5010-fed mice following infection with the R20291 strain, recapitulating the phenotype observed following VPI10463 infection (Fig. S2A through C).

Diet-mediated differences in disease severity were independent of *C. difficile* burden in the large intestine as both Diet 5010-fed and Diet 5053-fed mice established comparable burden in the cecum at day 2 p.i. (Fig. 1D). *C. difficile* produces two toxins, Toxin A and B, that damage the intestinal epithelium as its primary virulence factors (2, 3). Similar to *C. difficile* burden, toxin production in the large intestine was not different between mice fed Diet 5010 or 5053 (Fig. 1E). Despite no differences in toxin production, histological examination of the proximal colon from Diet 5053-fed mice showed less intestinal damage and tissue pathology (Fig. 1F and G), indicating Diet 5053 may protect by maintaining host intestinal epithelial integrity. Breakdown of the composite histological score showed higher epithelial cell loss and intraluminal exudate in Diet 5010-fed mice (Fig. 1H). To corroborate Diet 5053-mediated protection of the intestinal epithelium, albumin and lipocalin-2 were measured in the cecal luminal content at day 2 p.i. Albumin in the intestinal content can be indicative of protein loss or bleeding due to barrier permeability, which can be a hallmark of *C. difficile* pathology (37, 38), while lipocalin-2 is commonly used as a marker of intestinal inflammation (39). Mice fed Diet 5053 had significantly less albumin (Fig. 1I) and lipocalin-2 (Fig. 1J) in the cecal content at day 2 p.i. compared to Diet 5010-fed mice, indicating less intestinal tissue damage and inflammation during infection. Taken together, these data suggest Diet 5053 protects mice from severe *C. difficile* infection by limiting damage to the host intestinal epithelium.

### Diet 5010 and Diet 5053 result in different magnitudes of inflammatory immune response during infection

**Fig 2:**
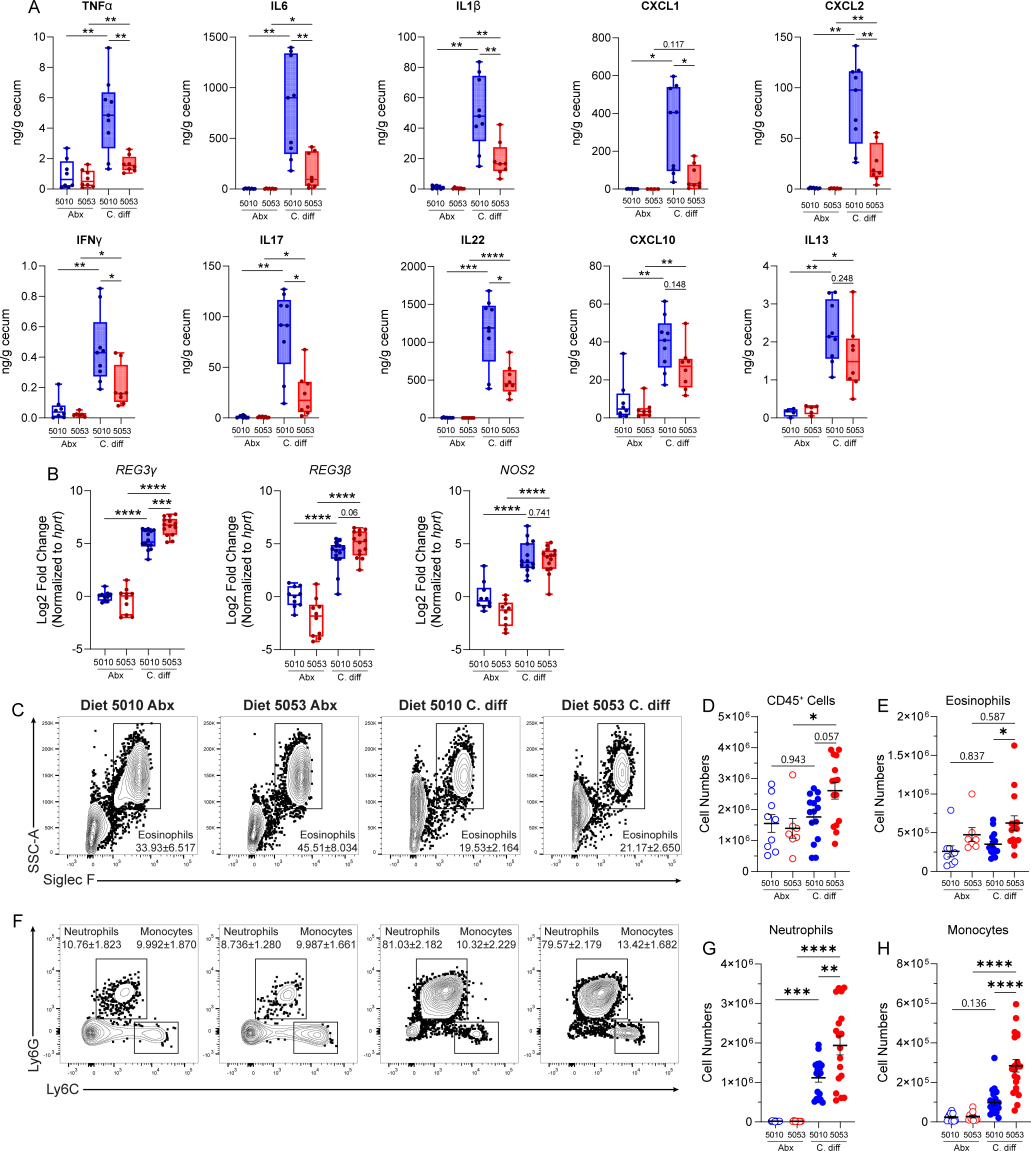
Diet 5010 and Diet 5053 result in differential magnitudes of immune response to *C. difficile*. (A) Cytokine or chemokine concentrations in intestinal tissue of mice; for Diet 5010 Abx*, n* = 7; Diet 5053 Abx*, n* = 7; Diet 5010 CDI*, n* = 7; Diet 5053 CDI*, n* = 8. (B) Gene expression in proximal colon tissue of mice before and during *C. difficile* infection relative to Abx-treated, uninfected 5010-fed mice. Gene expression was normalized to *Hprt*. Diet 5010 and Diet 5053 Abx groups*, n* = 10; for Diet 5010 and 5053 CDI groups*, n* = 14. (C) Frequencies of eosinophils in the colonic lamina propria at day 2 p.i. Fluorescence-activated cell sorting (FACS) plots gated on to live, CD45^+^, non-B, non-T cells. (D) Numbers of CD45^+^ cells in the colonic lamina propria at day 2 p.i. (E) Numbers of eosinophils in the colonic lamina propria. (F) Frequencies of neutrophils and monocytes in the colonic lamina propria FACS plots gated on to live, CD45^+^, non-B, non-T, Siglec-F^Neg^ cells. (G) Numbers of eosinophils in the colonic lamina propria. (H) Numbers of monocytes in the colonic lamina propria. For panels C–H: Diet 5010 Abx (*n* = 9), Diet 5053 Abx (*n* = 8), Diet 5010 CDI (*n* = 15), Diet 5053 CDI (*n* = 15). Data are mean ± SEM. Data in panels A and B were analyzed by *t*-test with false discovery rate (FDR) correction; data in C–H were analyzed by one-way analysis of variance with Tukey’s *post hoc* multiple comparisons test. * = *P* < 0.05; ** = *P* < 0.01; *** = *P* < 0.001; **** = *P* < 0.0001.

*C. difficile*-mediated damage to the intestinal epithelial barrier leads to a downstream inflammatory immune response. The acute immune response can include secretion of proinflammatory cytokines and chemokines leading to migration and activation of innate immune cell subsets in the large intestine lamina propria (LP). To define the intestinal immune response between Diet 5010- and Diet 5053-fed mice during acute infection, mice were sacrificed at day 2 p.i. and induction of immune mediators and recruitment of innate immune cells into the large intestine were assessed. Both protein quantification and gene expression showed a less inflammatory environment in the large intestinal tissue of mice fed Diet 5053 compared to mice fed Diet 5010 following infection (Fig. 2A; Fig. S3A). Specifically, diminished induction of tumor necrosis factor alpha (TNF-α), interleukin 6 (IL-6), IL-1β, C-X-C chemokine ligand 1 (CXCL1), CXCL2, and CXCL10 was observed in Diet 5053-fed mice following infection, all of which are inflammatory mediators associated with worse *C. difficile* infection outcomes (40–44) (Fig. 2A; Fig. S3A) and corroborate findings of a reduced inflammatory intestinal environment as evidenced by histologic examination (Fig. 1F and G) and lipocalin-2 levels (Fig. 1I). Production of interferon gamma (IFN-γ), IL-17a, and IL-22 was also significantly blunted in Diet 5053-fed mice at day 2 p.i. (Fig. 2A), despite these cytokines previously being linked to host protection (45–47). Despite reduced production of both beneficial and detrimental inflammatory mediators, Diet 5053-fed mice exhibited comparable or increased expression of the anti-bacterial host defense genes Reg3β, Reg3γ, and Nos2 (Fig. 2B). Taken together, this immune profile indicates that Diet 5053-fed mice are responding to *C. difficile* infection, but the magnitude of the response is more limited, potentially due to a decrease in observed epithelial damage (Fig. 1F and G).

Next, the innate immune cellular response in the lamina propria during infection was profiled by flow cytometric analysis (Fig. S4A and B). Previous work has found that innate immune cell subsets in the intestine, including innate lymphoid cells (ILCs), neutrophils, and eosinophils, play protective roles during *C. difficile* infection (25, 41, 48–61). Based on the gene expression profile and cytokine levels (Fig. 2A and B; Fig. S3A), we predicted that the cellular response to infection would similarly be reduced in Diet 5053-fed mice. However, no difference was observed in the proportion or numbers of IFN-γ-producing ILCs (ILC1s) or IL-22-producing ILCs (ILC3s) between Diet 5010- and Diet 5053-fed mice following infection (Fig. S5A through C). There were increased absolute numbers of eosinophils (Fig. 2E), neutrophils (Fig. 2G), and inflammatory monocytes (Fig. 2H) in the lamina propria of Diet 5053-fed mice, although the relative frequencies of these cells were similar between Diet 5010- and Diet 5053-fed mice (Fig. 2C and F). The increased numbers of these immune cell subsets are driven by increased infiltration of CD45^+^ cells in the lamina propria of Diet 5053-fed mice during infection (Fig. 2D). Collectively, innate immune profiling suggests a more controlled inflammatory response in Diet 5053-fed mice compared to Diet 5010-fed mice. The innate immune response in Diet 5053-fed mice is indicative of a host response that is limiting infection-mediated pathology without immune-driven collateral damage.

### Diet-mediated protection from severe infection is dependent on the microbiota

**Fig 3:**
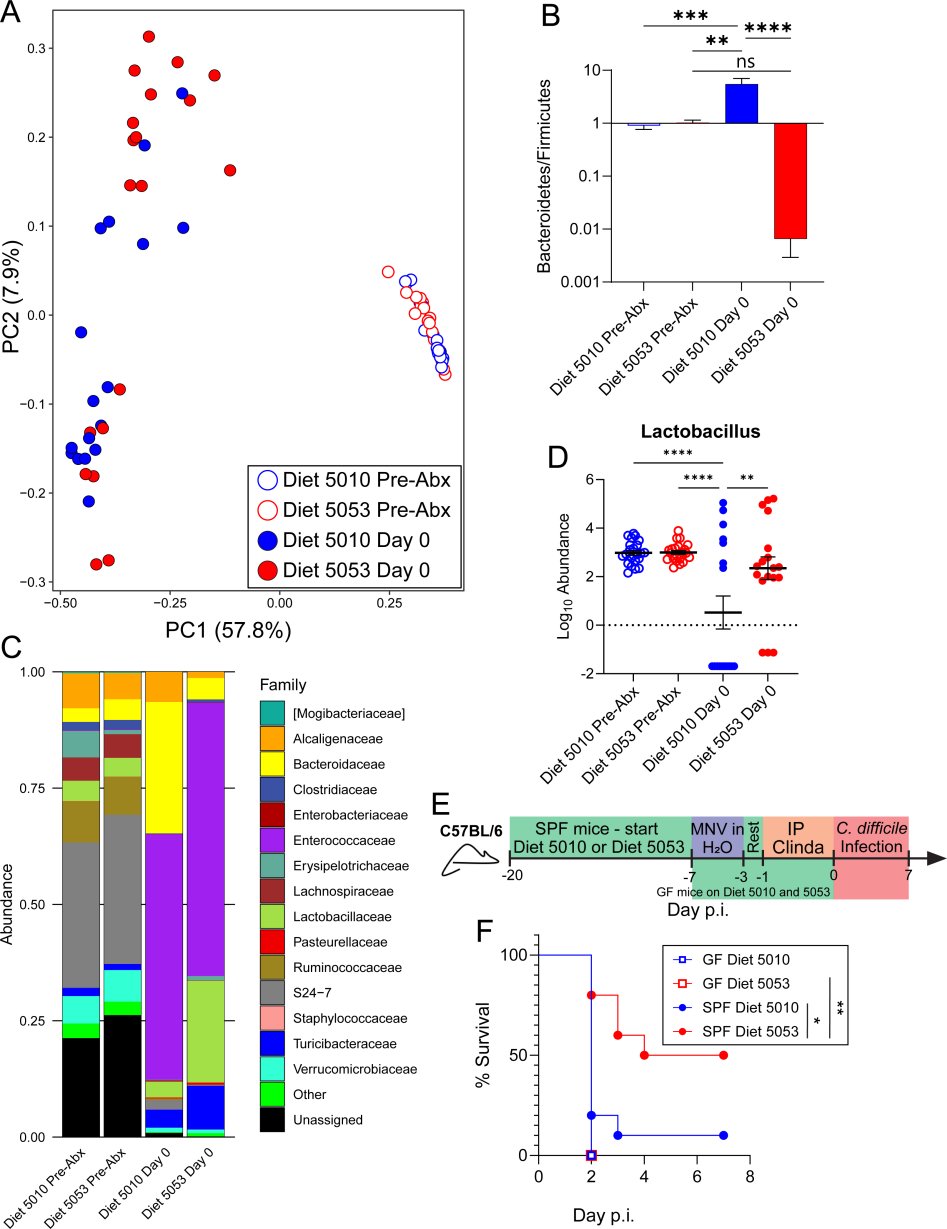
Susceptibility to severe *C. difficile* infection is not imprinted on the host. (A) Principal coordinate analysis of unweighted UniFrac distances of mice fed Diet 5010 or Diet 5053 before and after antibiotic treatment. (B) The Bacteroides/Firmicutes ratio of ASV counts from mice fed Diet 5010 or Diet 5053 before and after antibiotic treatment. (C) Relative abundance of the top 15 taxonomic families of mice fed Diet 5010 or Diet 5053 before and after antibiotic treatment. (D) Differential abundance of a *Lactobacillus* ASV before and after antibiotic treatment. (E) Experimental design of germ-free diet experiments. (F) Survival of germ-free mice and SPF control mice during infection. For all groups in panels A–D, *n* = 19 mice; for all groups in F, *n* = 10 mice. Data in panels B and D are mean ± SEM. Data in panels B and D were analyzed by one-way analysis of variance with Tukey’s *post hoc* multiple comparisons test; data in panel F were analyzed by the Mantel-Cox (log rank) test with Holm-Sidak correction. * = *P* < 0.05; ** = *P* < 0.01; *** = *P* < 0.001; **** = *P* < 0.0001.

Diet is a major driver of intestinal microbiota composition (10), which may then affect the host in a variety of ways, from immune function to metabolic status (62). To determine if Diet 5053-fed mice have a distinct microbiota composition from Diet 5010-fed mice, 16S rRNA sequencing was performed on fecal pellets before and after antibiotic treatment prior to infection. Mice fed Diet 5010 or Diet 5053 had similar intestinal microbiota compositions after 2 weeks on different diets (Fig. 3A). Antibiotic treatment prior to *C. difficile* infection led to the microbiota compositions of Diet 5010- and Diet 5053-fed mice shifting and becoming more variable (Fig. 3A). One of the largest changes after antibiotic treatment in the microbiota compositions between Diet 5010- and Diet 5053-fed mice was the ratio of Bacteroides and Firmicutes phyla increasing in Diet 5010-fed mice but decreasing in Diet 5053-fed mice, respectively (Fig. 3B). Comparing the relative abundance of bacterial families revealed that Diet 5053-fed mice have increased abundance of Lactobacillaceae after antibiotic treatment (Fig. 3C), and the *Lactobacillus* ASV driving this difference was maintained in Diet 5053-fed mice after antibiotic treatment (Fig. 3D). Members of this family are often associated with probiotic or beneficial effects on the host, such as interacting with the aryl hydrocarbon receptor to improve colitis or reducing *C. difficile* shedding (63, 64). While these observations show that changes in the microbiota composition between Diet 5010-fed and Diet 5053-fed mice occur after antibiotic treatment, the contribution of the microbiota to diet-mediated protection was unclear. To test whether diet-mediated protection is dependent on the intestinal microbiota, age-matched germ-free mice were placed on Diet 5010 or Diet 5053 for 7 days prior to *C. difficile* infection alongside conventional specific pathogen-free (SPF) mice (Fig. 3E). Germ-free (GF) mice lack a microbiota and therefore serve to decouple dietary effects directly on the host from indirect effects via the microbiota. Germ-free mice rapidly succumbed to *C. difficile* infection regardless of diet (Fig. 3F), while SPF mice fed Diet 5053 were protected from severe disease (Fig. 3F), indicating that the microbiota is necessary for diet-mediated protection during *C. difficile* infection.

### Diet-mediated protection requires a specific initial microbiota composition and is not imprinted on the host

**Fig 4:**
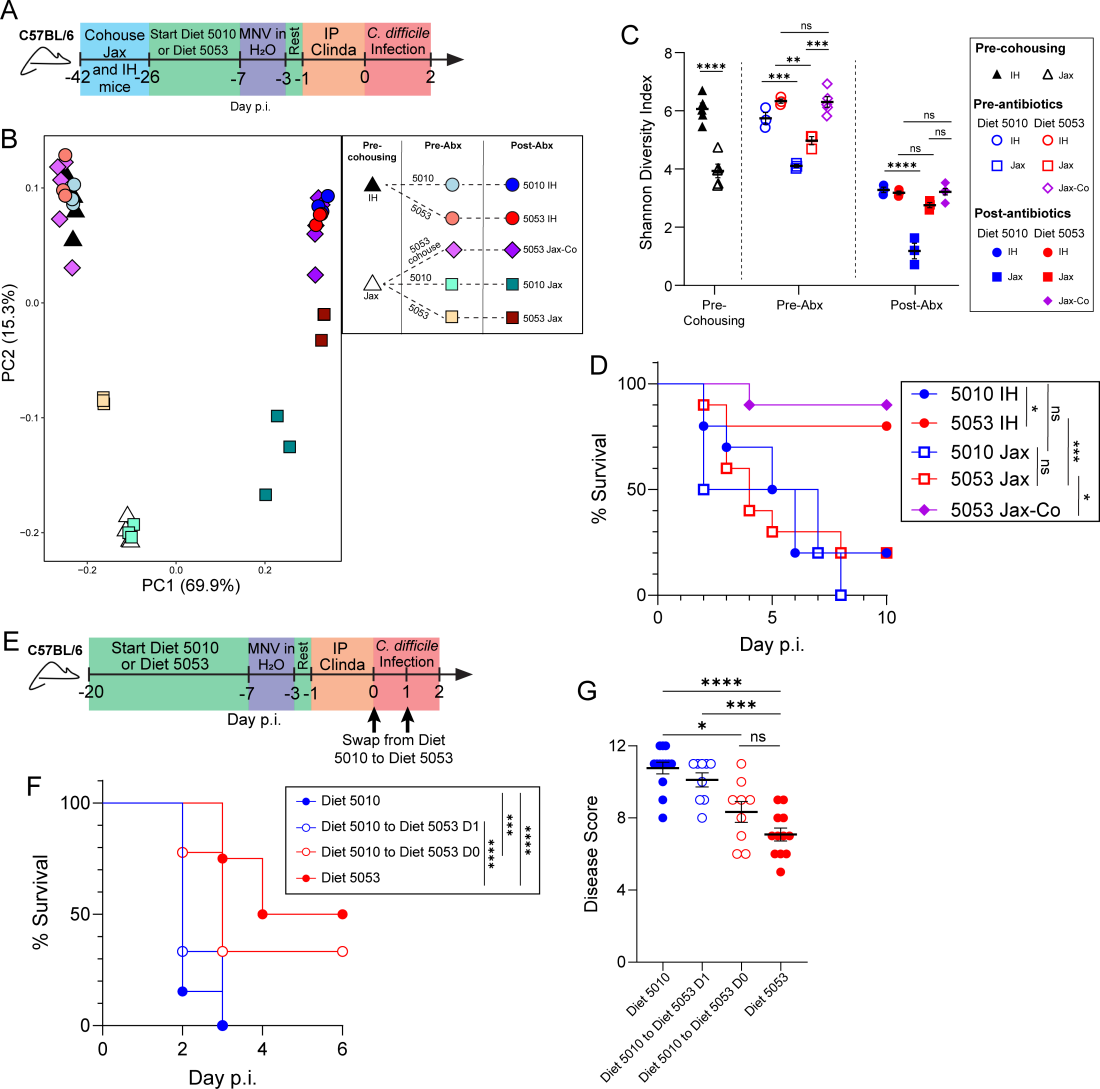
Diet-mediated protection from severe *C. difficile* infection is dependent on the gut microbiota composition. (A) Cohousing experimental design. (B) Principal coordinate analysis of weighted UniFrac distances. (C) Alpha diversity of groups using the Shannon Diversity Index. (D) Survival of IH, Jax, and cohoused Jax (Jax-Co) C57BL/6 mice following infection. (E) Diet swap experimental design. (F) Survival of mice swapped from Diet 5010 to Diet 5053. (G) Disease severity of mice swapped from Diet 5010 to Diet 5053. For panels B and C: pre-cohoused groups (*n* = 5), pre-antibiotic cohoused Jax (*n* = 5), pre-antibiotic non-cohoused IH (*n* = 3), post-antibiotic cohoused Jax (*n* = 5), post-antibiotic non-cohoused IH (*n* = 3). For panel D: all groups (*n* = 10). For panels F and G: Diet 5010 (*n* = 13), Diet 5010 to Diet 5053 D1 (*n* = 9), Diet 5010 to Diet 5053 D0 (*n* = 9), Diet 5053 (*n* = 12). Data in panels D and F were analyzed by the Mantel-Cox (log rank) test with Holm-Sidak correction; data in panels C and G were analyzed by one-way analysis of variance with Tukey’s *post hoc* multiple comparisons test. * = *P* < 0.05; ** = *P* < 0.01; *** = *P* < 0.001; **** = *P* < 0.0001.

Germ-free mice completely lack a microbiota and are acutely susceptible to *C. difficile* infection (27, 65). Therefore, the contribution of the microbiota to Diet 5053-mediated protection was further tested in mice with different initial microbiota composition. Mice bred in different institutions or sourced from different vendors have distinct intestinal microbiota compositions (66–71). For example, C57BL/6 mice from the Jackson Labs maximum barrier facility have a less diverse microbiota than other vendors (66, 72). Age-matched Jackson C57BL/6 mice (Jax) were fed Diet 5010 or Diet 5053, housed in separate cages, and infected with *C. difficile* along with C57BL/6 mice bred in-house (IH) in the vivarium at the University of Pennsylvania (Fig. 4A). Jax mice started with a distinct microbiota composition (Fig. 4B) and reduced alpha diversity (Fig. 4C) compared to IH mice, and the microbiota compositions remained distinct following the start of each diet and following antibiotic treatment (Fig. 4B and C). Following infection, both Diet 5010- and Diet 5053-fed groups of Jax mice succumbed to infection while Diet 5053-fed IH mice exhibited significantly reduced mortality (Fig. 4D). In contrast, Jax mice that were cohoused with IH Diet 5053-fed mice prior to antibiotic treatment and infection (Fig. 4A) displayed an increase in alpha diversity following cohousing (Fig. 4C) and the microbiota composition shifted similarly to the IH mouse microbiota composition (Fig. 4B). Following equilibration of the microbiota, cohoused Jax mice fed Diet 5053 exhibited increased survival following *C. difficile* infection (Fig. 4D). To determine if Diet 5053-mediated protection was unique to mice raised in the University of Pennsylvania vivarium, C57BL/6 mice from Charles River Laboratories were fed Diet 5010 or Diet 5053 and infected with *C. difficile* VPI10463 without cohousing with IH C57BL/6 mice. Despite coming from an outside vendor, these mice displayed infection severity phenotypes similar to Diet 5010- and Diet 5053-fed C57BL/6 mice bred at the University of Pennsylvania (Fig. S6A through C). These results provide evidence that Diet 5053 alone is not sufficient to convey protection but requires a specific, potentially more diverse microbiota to protect mice from *C. difficile* infection.

Since a specific microbe or consortia of microbes breaking down dietary components appears to be required for protection, one question that follows is whether the diet-mediated susceptibility phenotypes are long-lasting in the host following weeks of diet consumption or if susceptibility to *C. difficile* infection is malleable with rapid dietary intervention strategies. To address this question, mice were fed Diet 5010 but had their feed swapped to Diet 5053 on day 0 or 1 p.i. (Fig. 4E). Similar to mice that had been fed Diet 5053 for 3 weeks prior to infection, mice that had their feed swapped to Diet 5053 as late as the day of infection (day 0 p.i.) displayed a significant increase in survival and decrease in disease severity compared to Diet 5010-fed mice (Fig. 4F and G). Swapping from Diet 5010 to Diet 5053 at day 1 p.i. had no effect, as those mice had no difference in phenotype compared to the Diet 5010 controls (Fig. 4F and G). Because of the malleability of the diet phenotype, we concluded that susceptibility to severe disease is not imprinted on the host and highlights the potential clinical relevance for an intervention strategy for at-risk patients.

### Changes in microbiota metabolic function are associated with protection from severe disease

**Fig 5:**
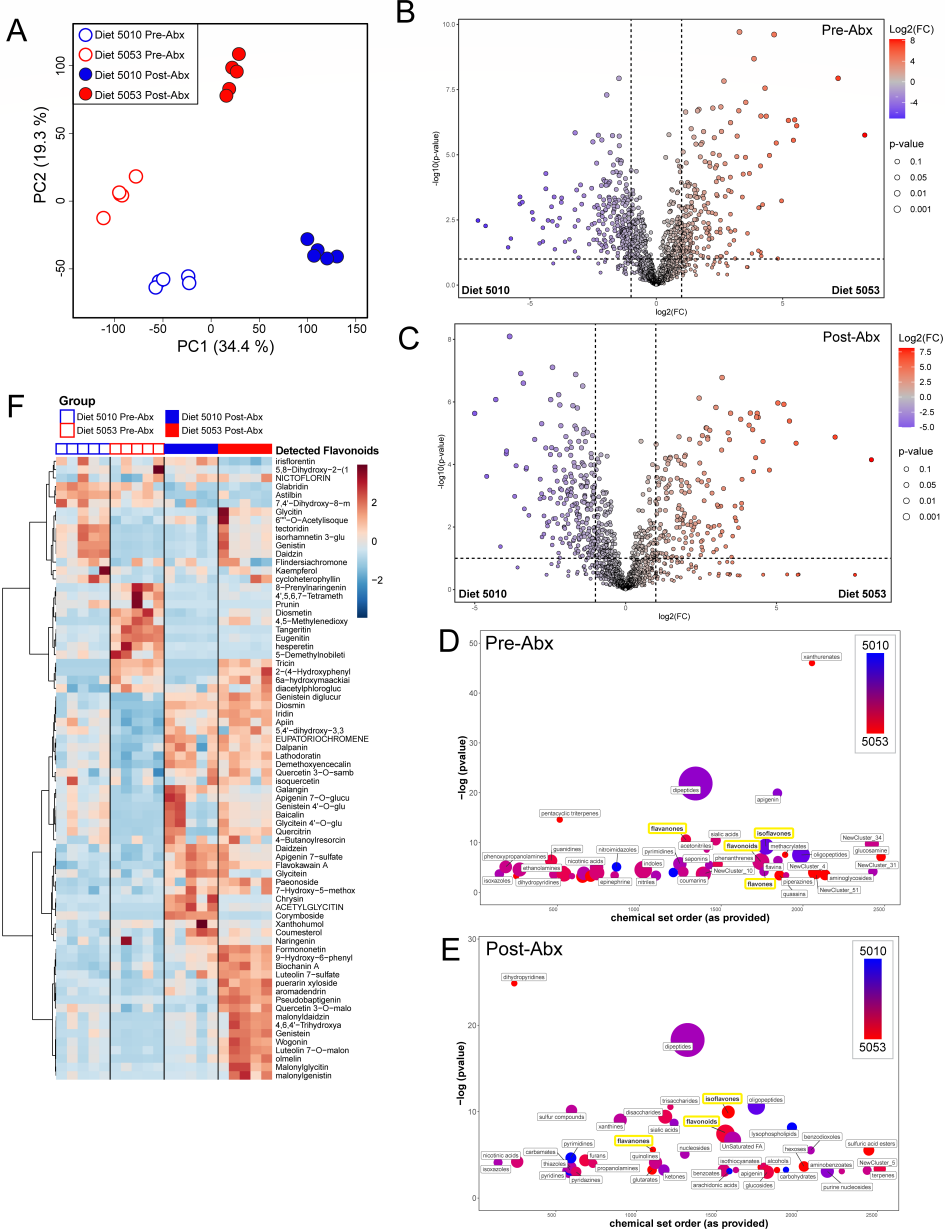
Diet 5010 and Diet 5053 differentially modify microbiota function before and after antibiotic treatment. (A) Principal component analysis of intestinal metabolomes from mice fed Diet 5010 or Diet 5053 before and after antibiotic treatment. (B) Volcano plot showing differentially abundant metabolites before and (C) after antibiotic treatment. (D) ChemRICH plot identifying enriched classes of metabolites before antibiotic treatment or (E) after antibiotic treatment. (F) Heatmap of detected flavonoid metabolites before and after antibiotic treatment. For all groups, *n* = 5 mice.

The intestinal microbiota can interact with the host by producing biologically active metabolites. Depending on the intestinal microenvironment, the same microbiota compositions can adapt their metabolic output and may have very different functional properties and distinct influences on host physiology (73–75). Thus, we sought to characterize differences in metabolic function of the microbiota in mice fed Diet 5010 or Diet 5053. We performed targeted metabolomics on bile acids, amino acids, and short-chain fatty acids, metabolite classes associated with *C. difficile* infection and metabolism (76–78). While there were major differences after antibiotic treatment compared to before (Fig. S7A), there were few differences between Diet 5010- and 5053-fed mice at either time point (Fig. S7A). There were no significant differences in primary or secondary bile acids (Fig. S7B and C). After antibiotic treatment, there were increased concentrations in the short-chain fatty acids (isobutyrate and propionate) and amino acid (proline), known to alter *C. difficile* growth kinetics (73–75), in Diet 5010-fed mice (Fig. S7D and E). However, examination of *C. difficile* early burden (12 and 24 hours p.i.) revealed no difference in *C. difficile in vivo* growth kinetics in mice fed the two diets (Fig. S7F). Therefore, we performed a more comprehensive untargeted metabolite analysis on intestinal content from mice fed Diet 5010 or Diet 5053 before and after antibiotic treatment to reveal functional metabolic differences. Despite the similarity of the intestinal microbiota compositions of Diet 5010- and Diet 5053-fed mice before antibiotic treatment (Fig. 3A), the intestinal metabolomes of these mice were distinct at the same time point (Fig. 5A) and diverged further following antibiotic treatment (Fig. 5A). The distinct metabolome profiles before and after antibiotics were driven by many individual metabolites detected at significantly different levels at each time point (Fig. 5B and C), making it challenging to ascribe the protective capacity of Diet 5053 to one or a few specific metabolites. To further characterize metabolomic differences between Diet 5010- and Diet 5053-fed mice, chemRICH (79) was used to identify enrichment of metabolite classes at each time point (Fig. 5D and E). While there were many differences in metabolic classes between the Diet 5010 and Diet 5053 metabolomes before and after antibiotic treatment, one consistent shift was the enrichment and association of flavonoid classes in Diet 5053-fed mice, such as isoflavones and flavanones (Fig. S5D and E). Within the total detected flavonoids, Diet 5010- and 50530-fed mice have distinct compositions before antibiotic treatment, which are almost completely disrupted by antibiotic treatment (Fig. 5F). After antibiotic treatment, there appears to be a new shared “core” set of flavonoids enriched in both Diet 5010- and Diet 5053-fed mice, while Diet 5053-fed mice possess a unique set of flavonoids that are sparsely detected in any of the other groups (Fig. 5F). Flavonoids are associated with anti-inflammatory effects in mouse colitis models, and the microbiota plays a substantial role in metabolizing diet-derived flavonoids to increase bioavailability for the host (80–84). The metabolite profile of Diet 5053-fed mice is distinct from Diet 5010-fed mice and helps provide a biological explanation for the observed different susceptibility phenotypes to *C. difficile* infection.

### Diet 5053 protects mice from chemically induced colitis

**Fig 6:**
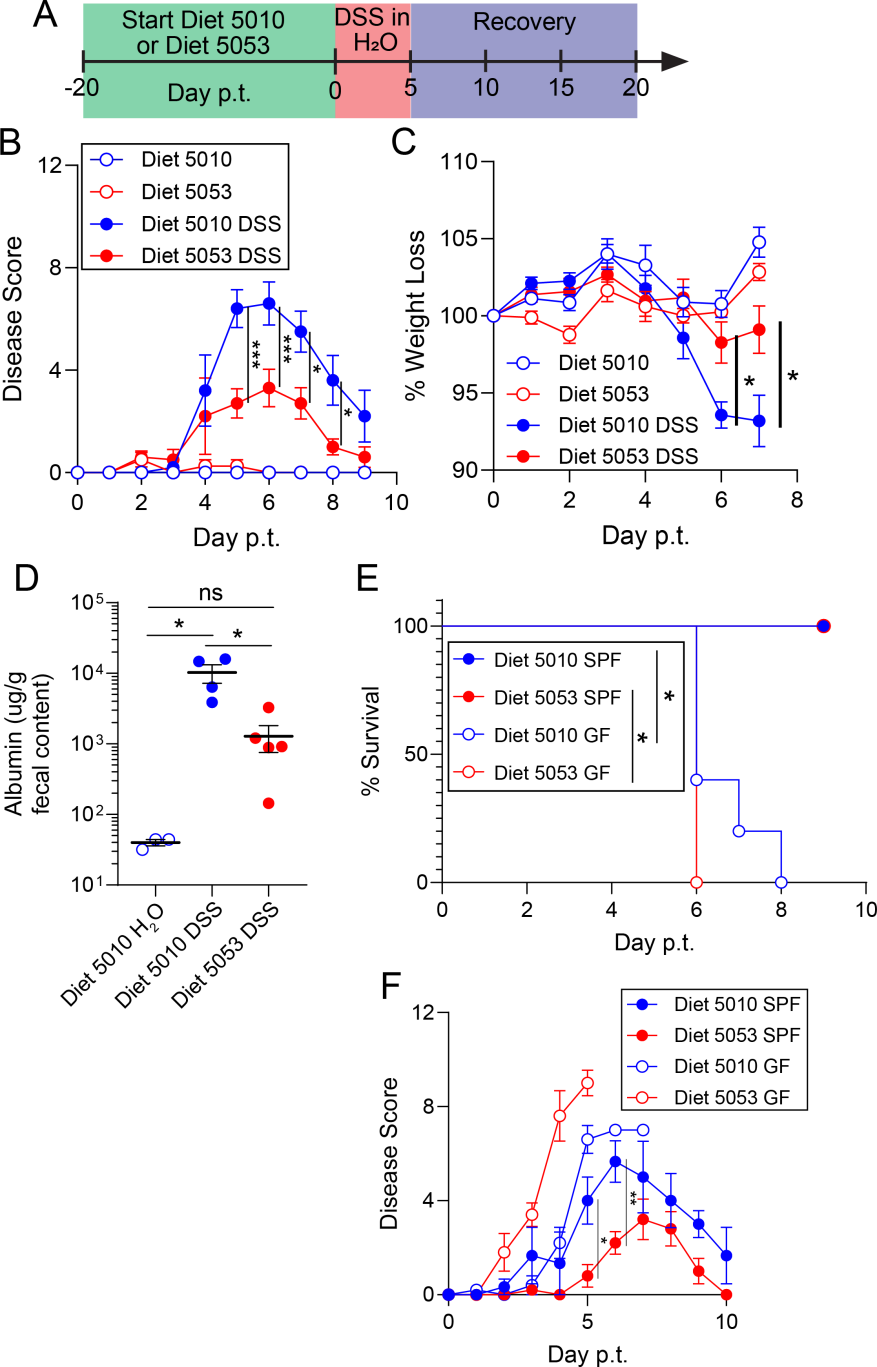
Diet 5053 protects mice from dextran sodium sulfate-induced colitis. (A) Experimental design for the DSS treatment model. (B) Disease severity of mice fed Diet 5010 or Diet 5053 and DSS-treated or untreated. (C) Weight loss of mice fed either Diet 5010 or Diet 5053 and DSS-treated or untreated. (D) Fecal albumin concentration at day 5 post-treatmet (p.t.) (E) Survival and (F) disease severity of germ-free and SPF mice treated with DSS. For panels B and C, Diet 5010 (*n* = 7), Diet 5053 (*n* = 4), Diet 5010 DSS (*n* = 10), Diet 5053 DSS (*n* = 10); for panel D, Diet 5010 (*n* = 3), Diet 5010 DSS (*n* = 4), Diet 5053 DSS (*n* = 5); for panels E and F, Diet 5010 SPF (*n* = 3), Diet 5053 SPF (*n* = 5), Diet 5010 GF (*n* = 5), Diet 5053 GF (*n* = 5). For panels B, C, D, and F, data are mean ± SEM and were analyzed by one-way analysis of variance with Tukey’s *post hoc* multiple comparisons; panel E was analyzed by the Mantel-Cox (log rank) test with Holm-Sidak correction. ns, not significant; * = *P* < 0.05; ** = *P* < 0.01; *** = *P* < 0.001.

Diet 5053-mediated protection is independent of *C. difficile* burden and toxin production (Fig. 1D and E), preserves the intestinal epithelial tissue (Fig. 1F and G), and induces a metabolite signature in the intestinal lumen that is suggestive of anti-inflammatory properties (Fig. 5D and F). Therefore, we hypothesized that Diet 5053-mediated protection may extend beyond *C. difficile* infection and may provide protection against pathologies driven by intestinal inflammation. To test this hypothesis, the dextran sodium sulfate (DSS) colitis model of IBD (85) was implemented in Diet 5010- and Diet 5053-fed C57BL/6 mice bred in the University of Pennsylvania vivarium (Fig. 6A). Following administration of 3% DSS in the drinking water, Diet 5053-fed mice had lower disease severity compared to Diet 5010-fed mice (Fig. 6B). Reduced weight loss in Diet 5053-fed mice was a major driver for the reduced disease severity (Fig. 6C). Diet 5053-fed mice also had less albumin detected in the feces at day 7 post-treatment compared to Diet 5010-fed mice, indicating a more intact epithelial barrier (Fig. 6D). To confirm the role of the microbiota in diet-mediated protection following DSS-induced colitis, germ-free mice and conventional SPF control mice were fed Diet 5010 or Diet 5053 prior to being administered 3% DSS in the drinking water. Regardless of diet, the GF mice developed severe colitis and succumbed to severe disease, while the SPF controls fed Diet 5053 had less colitis compared to the Diet 5010-fed SPF mice (Fig. 6E and F). These differences in DSS colitis severity between Diet 5010- and Diet 5053-fed mice recapitulate the differences observed during *C. difficile* infection. Taken together, Diet 5053 appears to be broadly protective in both infectious and non-infectious colitis models by maintaining intestinal epithelial barrier, thereby limiting downstream colitis severity.

## DISCUSSION

In this report, mice fed two standard mouse diets with comparable macro and micronutrient compositions have differential disease severity in two models of colitis. In both *C. difficile* infection and DSS colitis, Diet 5053 leads to better protection of the epithelial tissue compared to Diet 5010. Diet 5053 alone was not sufficient to directly convey protection, but rather, protection is co-dependent on a microbiota composition with the capacity to produce protective metabolites from Diet 5053. Deciphering the specific metabolites driving protection could yield potential clinically relevant therapeutic strategies for patients and targets for future research.

Several gaps in knowledge still exist in this model. While the immune response to *C. difficile* in mice fed Diet 5053 appears to be less inflammatory, it is unknown whether this response is due to active suppression of inflammation or less immune activation due to less intestinal damage and less exposure to toxin or damage-associated molecular patterns (86). Despite less intestinal damage in Diet 5053-fed mice during infection, innate immune cell infiltration into the colonic lamina propria is increased compared to Diet 5010-fed mice. Eosinophils can play a protective role during *C. difficile* infection (48, 49) alongside their more general roles in wound healing and maintaining intestinal homeostasis (87, 88). The role of neutrophils and monocytes during *C. difficile* infection is more complex (41, 43, 58–61, 89), with these subsets being important in preventing opportunistic bystander bacterial dissemination but also contributing to immunopathology. As we have observed previously in this *C. difficile* infection model, neutrophil infiltration alone into the lamina propria during infection does not worsen disease despite their inflammatory potential (42). To note, the strains we used, VPI10463 and R20291, are two distinct ribotypes but are both considered hypervirulent and cause severe disease in mice. Milder strains, which cause less severe disease in mice, may not show as stark diet-dependent differences in disease severity. While there still could be similar mechanisms at play within the microbiota or host, the overall muted disease severity induced by a milder strain may mask any diet-dependent protection observed in more virulent strains.

It remains to be determined what members or metabolic functions of the microbiota are key for protection from severe disease. While proportional differences were observed in the microbiota compositions of mice fed Diet 5010 or Diet 5053, there was still substantial similarity between the two microbiotas. Our initial targeted metabolomic screen of *C. difficile*-associated metabolites showed few differences between Diet 5010- and 5053-fed mice before and after antibiotic treatment, which in combination with the *C. difficile* burden and toxin data (Fig. 1D and E) led to the conclusion that the protective phenotype is not affecting *C. difficile* directly. Further untargeted metabolomics analysis revealed stark differences in the intestinal metabolome of Diet 5010- and 5053-fed mice before and after antibiotic treatment that indicate that the metabolic output of the microbiota can rapidly change based on diet and may be playing a more central role in protection. The resulting postbiotic metabolites could potentially act on a variety of targets, such as the epithelial barrier, the immune system, or peripheral organ systems. One potential caveat to the untargeted metabolomic data is the methodology used is more favorable to aqueous metabolites as opposed to more hydrophobic metabolites like fatty acids. Despite this, we were able to detect a wide range of metabolites, including nucleotides, sugars, and peptides, among others, as seen in Fig. 5D and E. Within this untargeted metabolomic data set, flavonoids emerged as one of the larger sets of metabolites that was differentially enriched between Diet 5010- and Diet 5053-fed mice both before and after antibiotic treatment. Flavonoids are associated with anti-inflammatory effects and immune modulation in colitis models, and the microbiota plays a substantial role in metabolizing diet-derived flavonoids to increase bioavailability for the host (80–84). For example, the isoflavone genistein increased in Diet 5053-fed mice after antibiotic treatment. Like other flavonoids, the commensal bacteria cleave the conjugated sugar from the glycone genistin to create genistein, which is more readily absorbed by the host (80, 81). Genistein, among other flavonoids, has previously been shown to increase survival of mice infected with *C. difficile* by reducing apoptosis of intestinal epithelial cells and the production of inflammatory cytokines (90, 91). It has also been shown that different *Lactobacillus* taxa can metabolize specific flavonoids which can benefit the host. For example, the flavonoids luteolin and icariin were observed to reduce inflammation and severity of DSS colitis in rats and mice, respectively, while increasing the abundance of *Lactobacillus* (92, 93). *Lactobacillus vaginalis* produces a beta-galactosidase that can cleave the glycone moiety from daidzin to create daidzein, which reduced acetaminophen hepatotoxicity (94). Lactobacillaceae can protect against *C. difficile* infection, although there has been variability in clinical trials (95–98), where *Lactobacillus* probiotics may help reduce antibiotic-associated diarrhea but lack robustness as primary treatments for *C. difficile* infection. *In vitro*, there have been multiple mechanisms by which specific Lactobacillaceae can inhibit *C. difficile*, including production of reuterin by *Limosilactobacillus reuteri* (99) or use of bile salt hydrolases to produce bile acids inhibitory to *C. difficile* growth (100). *In vivo* mouse studies also generally support the protective effect of specific Lactobacillaceae during infection (63, 100–102). Diet 5053 may be supporting the growth of Lactobacillaceae, although this has not been tested directly; however, the observation that Diet 5053-fed mice do have higher relative abundances of Lactobacillaceae even after antibiotic treatment may support a diet-intrinsic role.

We also predict that the differentially abundant flavonoid signature found before antibiotic treatment in the *C. difficile* infection system may be involved in Diet 5053-mediated protection observed in the DSS colitis system as well. In both systems, mice are divided into groups fed either Diet 5010 or 5053 for at least 2 weeks prior to any additional experimental manipulation. Thus, the starting conditions are identical for each model, and the metabolome data from pre-Abx mouse groups in the *C. difficile* system can be applied to the pre-DSS groups in the DSS model.

Finally, the ability of Diet 5053 to also protect mice from DSS-induced colitis indicates a broad mechanism of protection from intestinal inflammation independent of *C. difficile* infection. This finding represents future translational research opportunities to identify the diet components that are ameliorating intestinal inflammation via microbiota programming. The necessity of the microbiota in the Diet 5053 protection in both *C. difficile* infection and DSS colitis suggests that there may be a specific member or members of the microbiota that possess the functional metabolism responsible for protection. However, more research is needed to understand the mechanistic role of Diet 5053 and how these findings may apply more specifically to *C. difficile* infection and IBD in humans.

Overall, this work represents a step forward in understanding how diet can impact not only *C. difficile* infection severity but potentially intestinal inflammation more broadly. These results also emphasize the need to account for and report the type of animal feed used in experimental systems, as even two standard chows can lead to very different outcomes.

## MATERIALS AND METHODS

### Mice

C57BL/6 mice were either bred in-house or purchased from the Jackson Laboratory; all mice were bred and raised at the University of Pennsylvania unless specified as coming from the Jackson Laboratory or Charles River Laboratories. Mice purchased from either the Jackson Laboratory or Charles River Laboratories were acclimated in the vivarium for at least 1 week prior to use. Six- to 10-week-old mice were used for experiments. Conventional (non-germ-free) SPF C57BL/6 mice were maintained in autoclaved cages on either LabDiet 5010 mouse chow (Diet 5010) (35) or LabDiet 5053 mouse chow (Diet 5053 or PicoLab Rodent Diet 20) (36) and autoclaved water under SPF conditions at the University of Pennsylvania. Both male and female mice were used for experiments with the exception of cohousing, during which only female mice were used to avoid the possibility of fighting among cohoused male mice. Germ-free mice were generated and housed within the Penn Gnotobiotic Mouse Facility at the University of Pennsylvania and were maintained on either LabDiet 5010 mouse chow (Diet 5010) or LabDiet 5053 mouse chow (Diet 5053) and autoclaved water.

### Antibiotic pretreatment, *C. difficile* infection, and mouse monitoring

Antibiotic pretreatment was begun 1 week before *C. difficile* infection. Metronidazole (0.25 g/L), neomycin (0.33 g/L), and vancomycin (0.33 g/L) (MNV cocktail) were mixed into the drinking water for 4 days before removal. One day following cessation of MNV-treated antibiotic water, mice received 200 µg of clindamycin (Sigma) by intraperitoneal injection. Twenty-four hours later, mice received approximately 200 *C*. *difficile* spores (VPI10463 strain) via oral gavage. After infection, mice were monitored and scored for disease severity by four parameters: weight loss (>95% of initial weight, 0; 95% to 90% initial weight, 1; 90% to 80% initial weight, 2; <80% initial weight, 3), surface body temperature (>32°C, 0; 32°C to 30.5°C, 1; 30.5°C to 29.5°C, 2; <29.5°C, 3), diarrhea severity (formed pellets, 0; loose pellets, 1; liquid discharge, 2; no pellets/caked to fur, 3), morbidity (score of 1 for each symptom with a maximum score of 3; ruffled fur, hunched back, lethargy, and ocular discharge). Mice that exhibited severe disease, defined as a surface body temperature below 29.5°C or weight loss in excess of 30%, were humanely euthanized by CO_2_ displacement.

### Quantification of *C. difficile* burden

Fecal pellets or large intestinal content were resuspended in deoxygenated phosphate-buffered saline (PBS), and 10-fold dilutions were plated on brain-heart infusion (BHI) agar supplemented with yeast extract (5 g/L), taurocholate (10 mL of 10% solution), L-cysteine (10 mL of 10% solution), D-cycloserine (4 mL of 62.5 g/L stock), and cefoxitin (0.5 mL of 16 g/L stock) at 37°C in an anaerobic chamber (Coy Labs) overnight (103). Cecal supernatants were collected and frozen at −80°C for downstream analysis.

### Quantification of *C. difficile* toxin burden

Vero cells were seeded in 96-well plates at 1 × 10^4^ cells per well and incubated for 24 h at 37°C in 5% CO_2_. Cecal supernatants were added in 10-fold dilutions to the Vero cells (100 µL per well) and incubated overnight prior to removal, rinsing with PBS, and replacement with fresh media. The presence of *C. difficile* toxins A and B was confirmed by neutralization with antitoxin antisera (Techlab). The data are expressed as the log10 reciprocal value of the last dilution where cell rounding was observed. Cell morphological changes were observed after 18 h using a Nikon inverted microscope. The cytopathic effect was determined as rounded cells compared to the negative-control wells.

### Dextran sodium sulfate treatment and mouse monitoring

DSS was sterile filtered and added to the drinking water for a final concentration of 3%. Mice underwent DSS treatment for 5 days before being returned to normal drinking water. From the start of treatment, mice were monitored daily for disease severity. Disease severity was measured as a composite score of weight loss (0 points no loss, 1 point = 1%–5% weight loss, 2 points = 5%–10% weight loss, 3 points = 10%–15% weight loss, and 4 points = more than 15% wt loss), stool consistency (0 points = normal, 2 points = loose stool, 4 points = watery diarrhea), and bleeding (0 points = no bleeding, 2 points = blood in stool, 4 points = gross bleeding) (104).

### Isolation of cells from intestinal lamina propria

Single-cell lymphocyte suspensions were obtained from the large intestine LP. Briefly, the large intestine was cut longitudinally and washed out with PBS. Intestinal tissues were incubated at 37°C under gentle agitation in stripping buffer (PBS, 5 mM EDTA, 1 mM dithiothreitol, 4% fetal calf serum (FCS), 10 µg/mL penicillin/streptomycin) for 10 min to remove epithelial cells, then for another 20 min for removal of intraepithelial lymphocytes. The remaining tissue was digested with collagenase IV 1.5 mg/mL (500 U/mL), DNase 20 µg/mL in complete media (Dulbecco’s minimal essential media supplemented with 10% FCS, 10 µg/mL penicillin/streptomycin, 50 µg/mL gentamicin, 10 mM HEPES, 0.5 mM beta-mercaptoethanol, 20 µg/mL L-glutamine) for 30 min at 37°C under gentle agitation. Supernatants containing the LP fraction were passed through a 100 µm cell strainer followed by passage through a 40 µm cell filter to achieve a single-cell suspension.

### Flow cytometry

Isolated LP cells were counted followed by incubation for at least 4 h in complete media in the presence of brefeldin A at 37°C. Cells were then washed in PBS followed by staining with a cell viability dye for 20 min at room temperature (Invitrogen AQUA dye). Cells underwent Fc blocking using anti-mouse CD16/32 (clone 2.4G2; BD Biosciences) and rat IgG (Sigma) followed by staining of surface markers with fluorescently conjugated antibodies. LP samples were split into two groups and were stained for surface markers including either CD3, CD5, CD8, CD19, CD45, Siglec F, Ly6G, Ly6C, MHCII (I-Ab), CD103, CD11b, and CD11c for innate immunity or CD3, CD4, CD5, CD8, CD19, CD45, CD90, CD127, NK1.1, and Gr-1 for intracellular cytokine staining (Table 1). The innate immune-stained samples were followed by fixation in 4% paraformaldehyde and maintenance in FACS buffer until analysis. After surface staining, the intracellular cytokine staining samples were permeabilized using IC (intracellular) fixation buffer (Invitrogen) and intracellularly stained with fluorescently conjugated antibodies for IFN-γ, IL-17, and IL-22 (Table 1). During intracellular staining, cells were washed and maintained in eBioscience permeabilization buffer (Invitrogen). LP cells were then fixed in 4% paraformaldehyde and maintained in FACS buffer until run. Samples were run on an LSR-II flow cytometer (BD) and analyzed using FlowJo software version 10.7.1 (BD). Cell populations were calculated from total cells per tissue and as a percentage of parent populations.

**TABLE 1:** Antibodies used for flow cytometry experiments

Marker	Clone	Fluorochrome	Catalog no.	Dilution	Company
Ly6g	1A8	A700	127622	100	BioLegend
CD45	30-F11	A700	103128	100	BioLegend
CD170 (Siglec-F)	S17007L	APC	155508	200	BioLegend
IFN-g	XMG1.2	APC	17–7311-82	200	Invitrogen
CD4	RM4-5	BV605	100548	200	BioLegend
CD45	30-F11	BV605	103155	200	BioLegend
CD19	6D5	BV650	115541	200	BioLegend
NK1.1	PK136	BV785	108749	100	BioLegend
CD19	6D5	BV785	115543	200	BioLegend
CD90.2	53–2.1	eF450	48–0902-82	300	eBioscience
MHC-II (I-Ab)	AF6-120.1	eF450	48–5320-82	200	eBioscience
Ly6c	HK1.4	eF780	47–5932-82	200	eBioscience
IL17A	TC11-18H10.1	FITC	506908	300	BioLegend
CD103	2E7	FITC	11–1031-82	200	Invitrogen
IL-22	1H8PWSR	PE	12–7221-82	100	eBioscience
Gr-1	RB6-8C5	PE Dazzle 594	108452	300	BioLegend
CD11c	N418	PE-Cy7	25–0114-82	300	eBioscience
CD127	A7R34	PE-Cy7	25–1271-82	100	Invitrogen
CD3e	1452C11	PerCP Cy5.5	45–0031-82	200	eBioscience
CD5	53–7.3	PerCp Cy5.5	45–0051-82	300	eBioscience
CD8a	53–6.7	PerCp Cy5.5	45–0081-82	300	eBioscience
CD11b	M1/70	PETxred/eF610	61–0112-82	300	Invitrogen
Rat IgG1	EBRG1	FITC	11–4301-82	300	eBioscience
Rat IgG1	EBRG1	PE	12–4301-82	100	eBioscience
Rat IgG1	EBRG1	APC	17–4301-82	200	eBioscience

### Bacterial DNA extraction,16S rRNA sequencing, and analysis

DNA was extracted from cecal content via Qiacube (Qiagen) using the DNeasy Powersoil Pro kit (Qiagen) according to the manufacturer’s instructions. Extracted DNA was then delivered to the PennCHOP Microbiome Core for 16S rRNA sequencing. Amplicons of either the V4-V5 16S rRNA region or the V1-V2 16S rRNA region were amplified and sequenced using an Illumina MiSeq platform as described previously (105). Sequenced data were imported into QIIME2 (v.2020.2) (106) and denoised using the DADA2 plugin (107). Resulting data were taxonomically classified in QIIME2 using the q2-feature-classifier (108) classify-sklearn naïve Bayes classifier with a newly generated classifier against Greengenes 13_8 99% operational taxonomic unit (OTU) sequences (109). Phylogenetic trees were generated using mafft (110) and the q2-phylogeny plugin (111). Data were then imported into R 3.6.3 (112) for further analyses with phyloseq (v.1.30.0) (113) and visualization with ggplot2 (v.3.3.0) (114). Unweighted UniFrac (115) dissimilarity was calculated to generate principal coordinate analysis (PCoA) plots.

### Metabolomics sample collection and mass spectrometry analysis

#### Extraction of intestinal contents from mice

For targeted metabolomics, fecal pellets were collected from mice, frozen in dry ice/ethanol immediately after collection, and stored at −80°C. For bile acid and amino acid analysis, samples were suspended in methanol (5 µL/mg stool), vortexed for 1 min, and centrifuged twice at 13,000 × *g* for 5 min. For short-chain fatty acid analysis, samples were homogenized in volatile free fatty acid mix (5 µL/mg stool, AccuStandard) and centrifuged twice (13,000 × *g* for 5 min).

For untargeted metabolomics, large intestinal contents were frozen in dry ice/ethanol immediately after collection and stored at −80°C. To extract metabolites, samples were thawed on ice, and 100 mg of intestinal contents was transferred to a 2 mL Eppendorf tube. A 1000 µL aliquot of 80% methanol was added to the tube followed by vortexing. Then, 200 µL of homogenate was extracted with 800 µL of 80% methanol, vortexed, and centrifuged for 5 min at 18,000 × *g* at 4°C. An 800 µL aliquot of supernatant was filtered through a 0.22 µm syringe and stored at −80°C. For liquid chromatography/mass spectrometry (LC/MS) metabolomics, two separate 350 µL methanolic supernatants (one for reversed-phase C18 and one for hydrophilic interaction liquid chromatography (HILIC) were dried down under nitrogen in a 96-well plate at 45°C. In addition, pooled quality controls (QCs) were prepared by combining a 50 µL aliquot from each methanolic supernatant, and 4 × 350 µL aliquots were similarly dried down. The dried down extracts were reconstituted and vortexed in high performance liquid chromatography (HPLC) mobile phases (100 µL of 90:10 water/methanol for C18 HPLC and 200 µL of 50 acetonitrile for HILIC for untargeted LC/MS metabolomics.

#### Targeted LC/MS metabolomics

Bile acids were quantified using a Waters Acquity uPLC System with a QDa single quadrupole mass detector. The supernatants collected above were analyzed on an Acquity uPLC with a Cortecs UPLC C-18 + 1.6 mm 2.1 × 50 mm column. The flow rate was 0.8 mL/min, the injection volume was 4 µL, the column temperature was 30°C, the sample temperature was 4°C, and the run time was 4 min per sample. Eluent A was 0.1% formic acid in water; eluent B was 0.1% formic acid in acetonitrile; the weak needle wash was 0.1% formic acid in water; the strong needle wash was 0.1% formic acid in acetonitrile. The seal wash was 10% acetonitrile in water. The gradient was 70% eluent A for 2.5 min, 100% eluent B for 0.6 min, and then 70% eluent A for 0.9 min. The mass detection channels were +357.35 for chenodeoxycholic acid and deoxycholic acid; +359.25 for lithocholic acid; −407.5 for cholic, alpha-muricholic, beta-muricholic, gamma-muricholic, and omega-muricholic acids; −432.5 for glycolithocholic acid; −448.5 for glycochenodeoxycholic and glycodeoxycholic acids; −464.5 for glycocholic acid; −482.5 for taurolithocholic acid; −498.5 for taurochenodeoxycholic and taurodeoxycholic acids; and −514.4 for taurocholic acid. Samples were quantified against standard curves of at least five points run in triplicate. Standard curves were run at the beginning and end of each metabolomics run. Quality control checks (blanks and standards) are run every eight samples.

Amino acids were quantified using a Waters Acquity uPLC System with an AccQ-Tag Ultra C18 1.7 µm 2.1 × 100 mm column and a Photodiode Detector Array. Amino acids in the supernatants collected above were derivatized using the Waters AccQ-Tag Ultra Amino Acid Derivatization Kit (Waters Corporation) and analyzed using the UPLC AAA H-Class Application Kit (Waters Corporation) according to manufacturer’s instructions. Quality control checks (blanks and standards) were run every eight samples. All chemicals and reagents used were mass spectrometry grade.

Short-chain fatty acids were quantified using a Waters Acquity uPLC System with an HSS T3 1.8 µm 2.1 × 150 mm column and a Photodiode Detector Array. The supernatants collected above were filtered through 1.2, 0.65, and 0.22 µm filter plates (Millipore), and the filtrate was loaded into a total recovery vial (Waters Corporation) for analysis. The flow rate was 0.25 mL/min, the injection volume was 5 µL, the column temperature was 40°C, the sample temperature was 4°C, and the run time was 25 min per sample. Eluent A was 100 mM sodium phosphate monobasic, pH 2.5; eluent B was methanol; the weak needle wash was 0.1% formic acid in water; the strong needle wash was 0.1% formic acid in acetonitrile; and the seal wash was 10% acetonitrile in water. The gradient was 100% eluent A for 5 min, 70% eluent B from 5 to 22 min, and then 100% eluent A for 3 min. The photodiode array was set to read absorbance at 215 nm with 4.8 nm resolution. Samples were quantified against standard curves of at least five points run in triplicate. Standard curves were run at the beginning and end of each metabolomics run. Quality control checks (blanks and standards) were run every eight samples.

#### Untargeted LC/MS metabolomics

Reversed-phase C18 chromatography to retain and separate medium polarity to nonpolar metabolites in each study sample was performed on a Thermo Scientific Vanquish UHPLC. HILIC chromatography was performed to retain highly polar metabolites not retained by reversed-phase C18 chromatography. A Vanquish UHPLC coupled to an Orbitrap ID-X mass spectrometer was scanned from *m*/*z* 60–1,000 at a resolution of 120,000 to identify several thousand metabolites in each sample. Compound Discoverer was used to process the LC/MS metabolomic data to identify metabolites and determine statistical differences between study groups.

### Metabolomic data analysis

Metabolomic data were analyzed using Metaboanalyst 5.0 (116–118) to generate principal component analysis (PCA) plots and heatmaps. ChemRICH (December 2021 version) (79) was used to determine enrichment of metabolite groups clustered under Medical Subject Headings classes.

### Tissue RNA isolation, cDNA preparation, and qPCR

RNA was isolated from colon tissue using mechanical homogenization and TRIzol isolation (Invitrogen) according to the manufacturer’s instructions. cDNA was generated using the QuantiNova Reverse Transcriptase Kit (Qiagen) according to the manufacturer’s instructions. qPCR was performed on cDNA using both the Taqman FAST Advanced Master Mix (Applied Biosystems) with TaqMan gene expression assay probes (Applied Biosystems) and the QuantiNova SYBR Green PCR Kit (Qiagen) with Quantitect primer assays (Qiagen). Reactions were run on a QuantStudio 6 Flex (Applied Biosystems). Genes of interest were displayed in Log2-normalized arbitrary units relative to the expression of Hprt.

### Histology sectioning and pathology scoring

Intestinal tissues were fixed with 10% neutral buffered formalin, embedded in paraffin, and 5 µm sections were cut and stained with hematoxylin and eosin (H&E). H&E-stained colon tissue sections were blindly scored, and a combined score was generated based on a scale of unremarkable (0) to severe (4) in the categories immune cell infiltration, epithelial cell loss, crypt hyperplasia, and intraluminal exudate.

### Intestinal tissue homogenization and analysis

Large intestinal tissue was collected and placed into Tissue Extraction Reagent (Thermo Fisher Scientific) containing cOmplete ULTRA Protease Inhibitor tablets (MilliporeSigma). Tissue was then homogenized using mechanical disruption and centrifuged, and the supernatants were collected and frozen at −80°C. Supernatants were transported on dry ice to the Penn Human Immunology Core for Luminex analysis (R&D Systems).

### Enzyme-linked immunosorbent assays

Supernatants from intestinal content were used to quantify albumin and lipocalin-2 concentrations. Albumin was quantified using the mouse Albumin ELISA kit (Fortis Life Sciences) and lipocalin-2 was quantified using the DuoSet Lipocalin-2 ELISA kit (R&D Systems) according to the manufacturer’s protocols.

### Statistical analysis

Data were graphed and analyzed in R version 3.6.3 (112) and GraphPad Prism version 10.0.2 (GraphPad Software). Statistical tests used are listed in the figure legends. For all experiments, alpha was set to 0.05.

## Data Availability

16S rRNA sequencing data is deposited in the NCBI Short Read Archive as BioProject PRJNA1208488. Metabolomic data in are available in Table S2.

## SUPPLEMENTAL MATERIAL

Supplemental Figures mbio.03302-24-s0001.pdf Fig. S1-S7.

Table S1 mbio.03302-24-s0002.docx Macromolecule comparisons between diet 5010 and diet 5053.

Table S2 mbio.03302-24-s0003.xlsx Metabolomics data.

ASM does not own the copyrights to Supplemental Material that may be linked to, or accessed through, an article. The authors have granted ASM a non-exclusive, world-wide license to publish the Supplemental Material files. Please contact the corresponding author directly for reuse.
