# Supplementary material for: Standard mouse diets lead to differences in severity in infectious and non-infectious colitis
Source: mBio. 2025 Mar 24;16(4):e03302-24. doi: 10.1128/mbio.03302-24 (PMC11980566; doi:10.1128/mbio.03302-24)
Supplement: Supplemental Figures — Fig. S1-S7. [file mbio.03302-24-s0001.pdf]

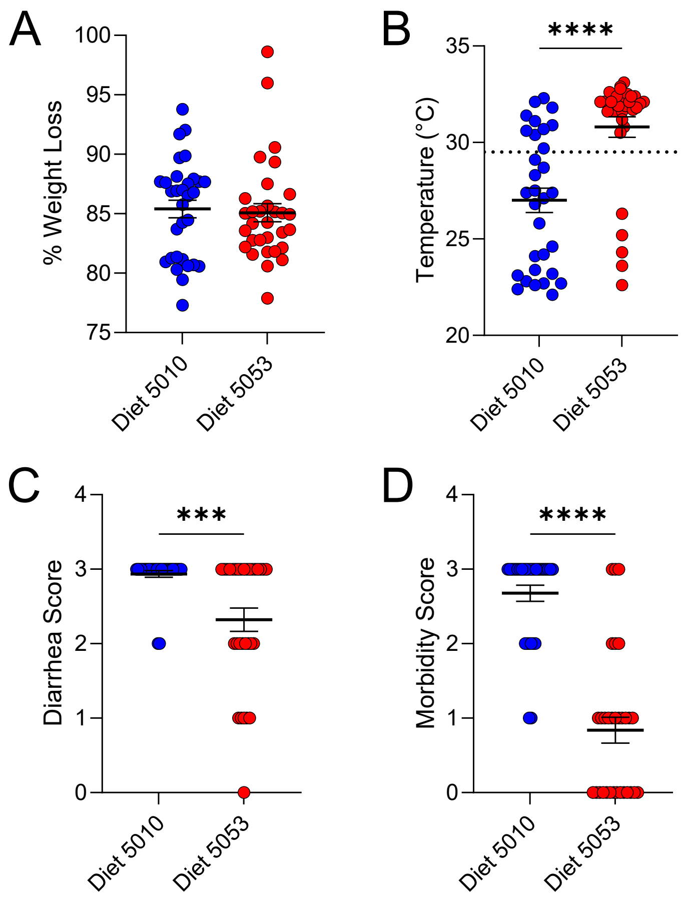

Supplementary Figure 1. Components of C. difficile disease severity score at day 2 p.i. A. Percent weight loss during *C. difficile* infection of mice fed Diet 5010 or Diet 5053. **B.** Body temperature. **C.** Diarrhea Score. **D.** Morbidity score. n=31 mice per group. Data are mean±SEM and were analyzed by t test. *** = p<0.001; **** = p<0.0001.

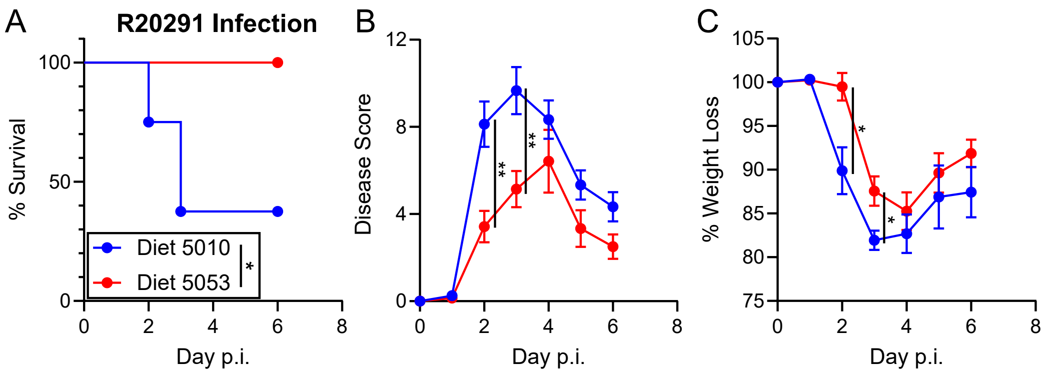

Supplementary Figure 2. Diet 5053 protects C57BL/6 mice from severe *C. difficile* infection with the R20291 strain of *C. difficile*. A. Survival of mice fed Diet 5010 or Diet 5053 after infection with strain R20291. **B.** Disease severity score during infection between mice fed Diet 5010 or Diet 5053. **C.** Weight loss during infection between mice fed Diet 5010 or Diet 5053. For Diet 5010-fed mice, n=8; for Diet 5053-fed mice, n=7. Data are mean±SEM. Data in **A** were analyzed by the Mantel-Cox (log rank) test, data in **B-C** were analyzed by t test at each time point. * = p<0.05; ** = p<0.01.

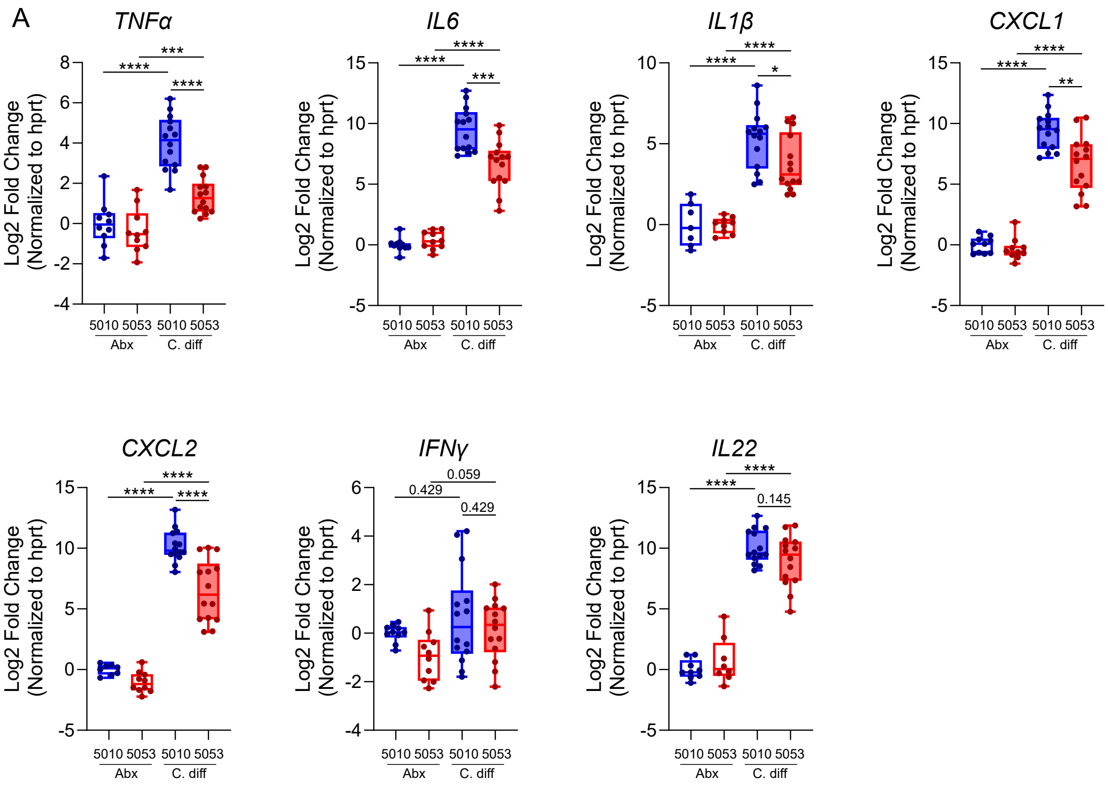

Supplementary Figure 3. Gene expression of cytokine and chemokine genes during *C. difficile* infection at day 2 p.i. A. Gene expression in proximal colon tissue of mice during *C. difficile* infection relative to antibiotic-treated, uninfected 5010-fed mice. Gene expression was normalized to Hprt. Diet 5010 and Diet 5053 Abx groups n=10, for Diet 5010 and 5053 CDI groups n=14. Data were analyzed by t test with FDR correction; * = p<0.05; ** = p<0.01; *** = p<0.001; **** = p<0.0001.

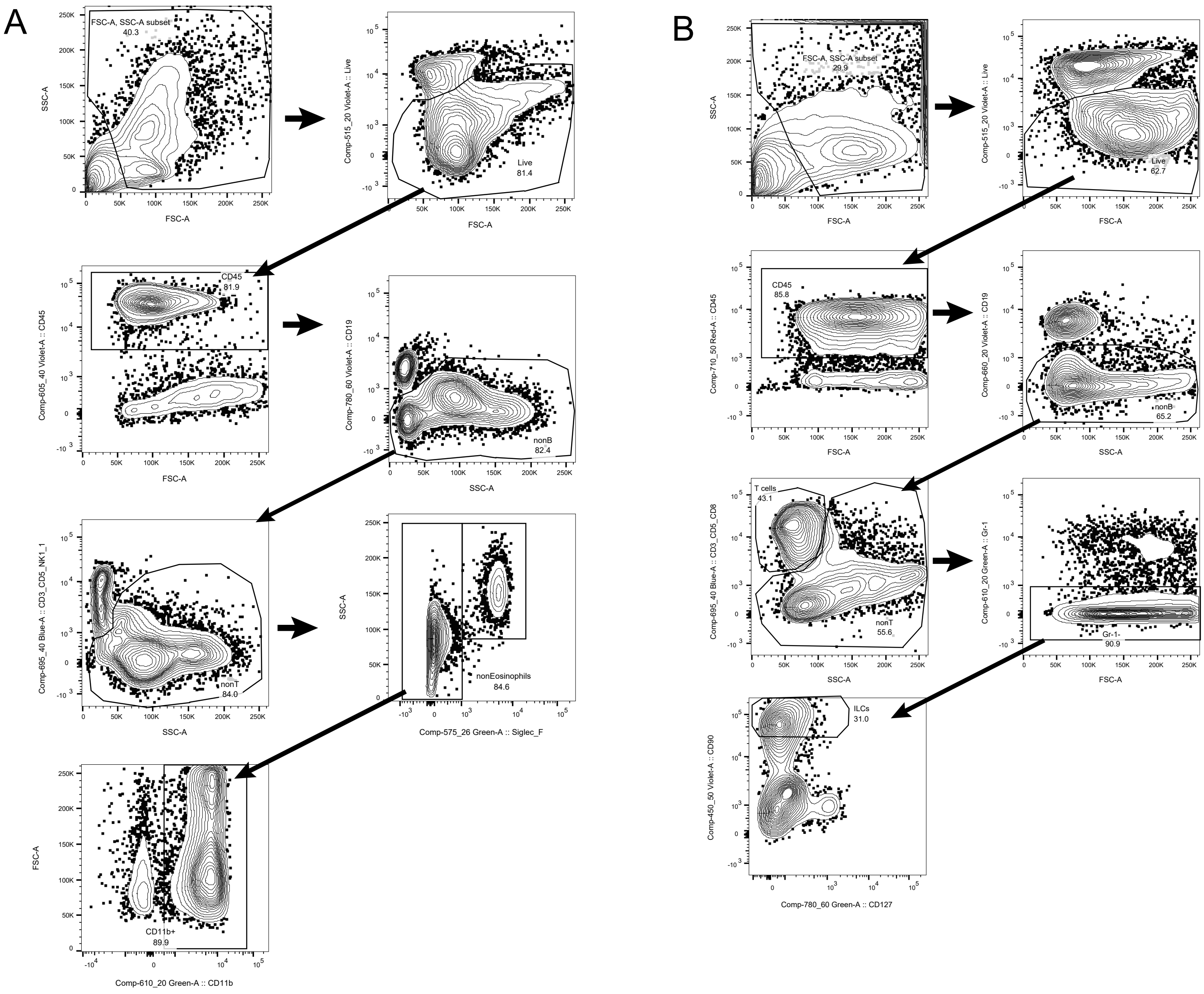

Supplementary Figure 4. Flow cytometry gating strategy for surface staining and intracellular cytokine staining. A. Representative gating for surface staining of innate immune cell populations, beginning with single cells to Live/Dead gating, CD45+ cells, non-B cells, non-T cells, non-eosinophils, to CD11b+ cells. **B.** Representative gating for surface staining of innate lymphoid cell populations, beginning with single cells to Live/Dead gating, CD45+ cells, non-B cells, non-T cells, Gr-1- cells, and CD90+ innate lymphoid cells.

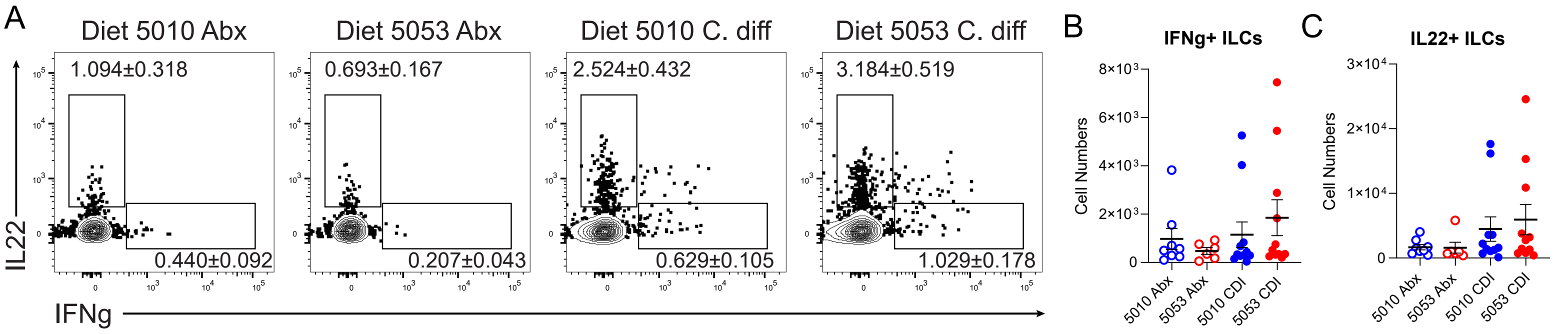

Supplementary Figure 5. Innate lymphoid cell gating and intracellular cytokine staining. A. Frequency and **B-C.** numbers of IFN-γ and IL-22-producing ILCs in the colonic lamina propria. Data are mean±SEM and were analyzed by one-way ANOVA with Tukey's post-hoc multiple comparisons.

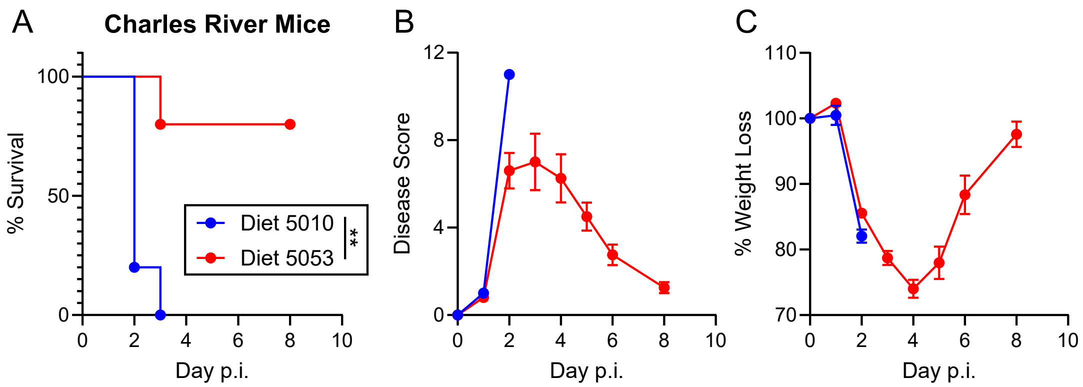

Supplementary Figure 6. C57BL/6 mice from a different source and fed Diet 5010 or Diet 5053 maintain differences in infection severity. A. Survival of Charles River C57Bl/6 mice fed Diet 5010 or Diet 5053. **B.** Disease severity score during infection between mice fed Diet 5010 or Diet 5053. **C.** Weight loss during infection between mice fed Diet 5010 or Diet 5053. For both groups, n=5. Data are mean±SEM. Data in **A** were analyzed by the Mantel-Cox (log rank) test. ** = p<0.01.

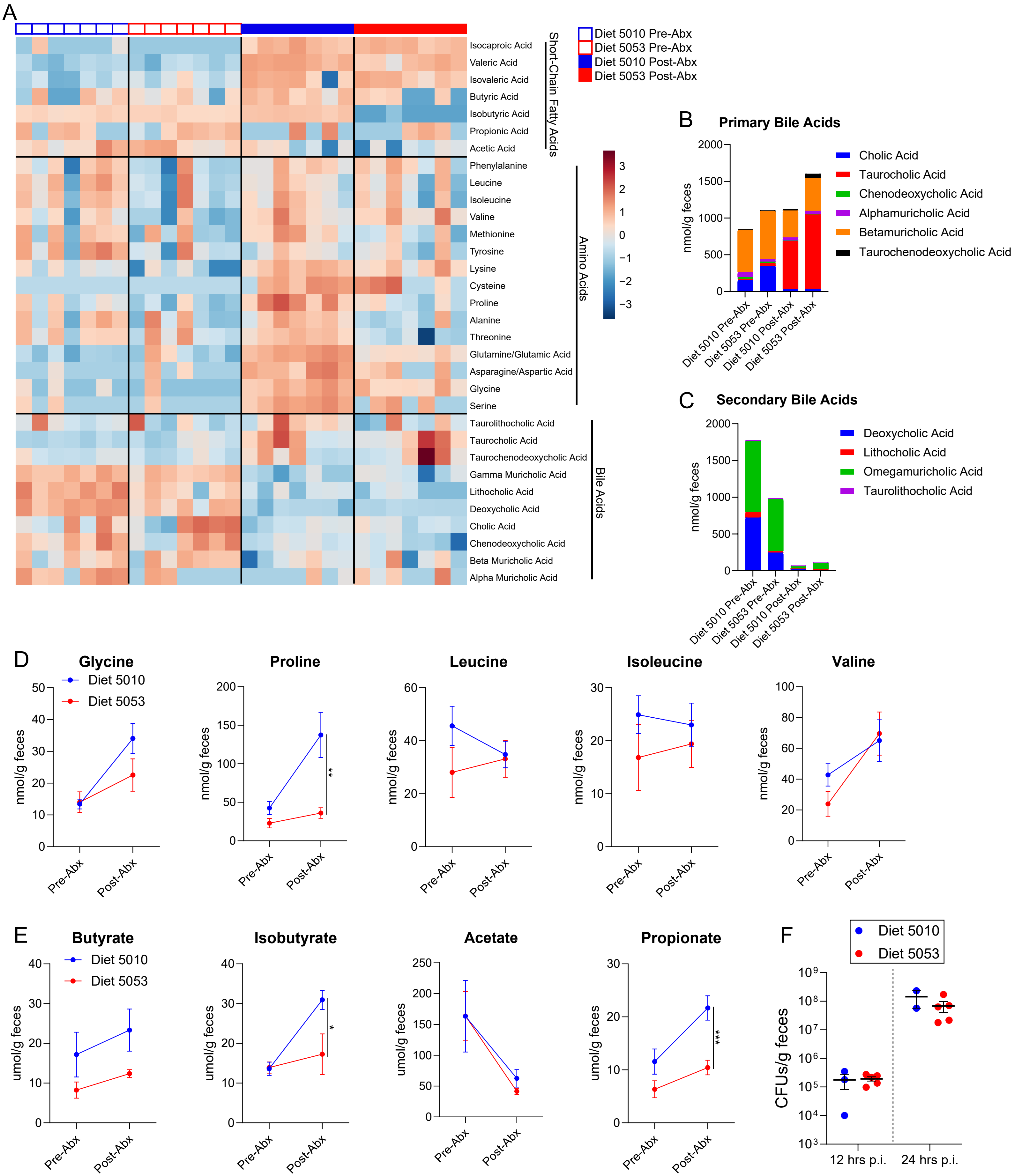

Supplementary Figure 7. Targeted metabolomics of *C. difficile*-associated metabolites reveal variability in Diet 5010-fed and Diet 5053-fed mice that do not affect infection kinetics. A. Heatmap of targeted metabolites measured in feces of C57Bl/6 mice before and after antibiotic treatment. The categories tested were short-chain fatty acids, bile acids, and amino acids and data was autoscaled for the heatmap. **B.** Concentrations of primary bile acids before and after antibiotic treatment. **C.** Concentrations of secondary bile acids before and after antibiotic treatment. **D.** Concentrations of specific amino acids related to *C. difficile*, including the branched chain amino acids. **E.** Concentrations of specific short-chain fatty acids. **F.** Measurement of *C. difficile* CFUs in the feces of mice by plating at 12 and 24 hours p.i.; *C. difficile* was undetected by plating before 12 hours p.i. and 1 Diet 5010-fed mouse was unable to produce a fecal pellet at 24 hours p.i. For all groups in **A-E**, n=7; for **F**, Diet 5010 n=3, Diet 5053 n=5. Data are mean±SEM. Data in **D-F** were analyzed by t test. * = p<0.05; ** = p<0.01.
