# Supplementary material for: Standard mouse diets lead to differences in severity in infectious and non-infectious colitis
Source: mBio. 2025 Mar 24;16(4):e03302-24. doi: 10.1128/mbio.03302-24 (PMC11980566; doi:10.1128/mbio.03302-24)
Supplement: Table S1 — Macromolecule comparisons between diet 5010 and diet 5053. [file mbio.03302-24-s0002.docx]

**Table S1.** Macromolecule comparisons between Diet 5010 and Diet 5053.

|  | **Diet 5010** | **Diet 5053** |
| --- | --- | --- |
| **Protein, %** | 24.6 | 21 |
| **Fat (Ether Extract), %** | 5 | 5 |
| **Carbohydrates (Starch+Sucrose), %** | 30.34 | 30.91 |
| **Fiber (Crude), %** | 4.2 | 4.4 |
| **Physiological Fuel Value, kcal/g** | 3.43 | 3.43 |
